# Using evidence-informed policies to tackle overweight and obesity in Chile

**DOI:** 10.26633/RPSP.2017.156

**Published:** 2017-12-19

**Authors:** Lorena Rodríguez Osiac, Cristian Cofré, Tito Pizarro, Cristián Mansilla, Cristian A Herrera, Jaime Burrows, Carmen Castillo

**Affiliations:** 1 Ministry of Health Ministry of Health Santiago Chile Ministry of Health, Santiago, Chile.

**Keywords:** Nutrition policy, obesity, legislation, food, food labeling, food publicity, Chile, Política nutricional, obesidad, legislación sobre alimentos, etiquetado de alimentos, publicidad de alimentos, Chile, Política nutricional, obesidade, legislação sobre alimentos, rotulagem de alimentos, publicidade de alimentos, Chile

## Abstract

*Overweight and obesity are a global epidemic with rates having risen to alarming levels in both developed and developing countries. Chile has been no exemption, with sharp increases in obesity prevalence, especially among school-age children. This paper describes the policy actions and strategies implemented to tackle this major public health concern in Chile over the last 10 years, and highlights the main challenges and nuances of the process. Chile has taken policy action that includes front-of-package labelling, advertising regulations, and school-food restrictions. New policies focus on the social determinants of health as they relate to food environments and people’s behavior. These actions are not only suitable to the current context in Chile, but are also supported by the best available scientific evidence. Moreover, the implementation of these policies has produced a broad debate involving public institutions and the food industry, with discussions issues ranging from property rights to trade barriers. Despite some differences among stakeholders, a valuable political consensus has been achieved, and several international organizations are eager to evaluate the impact of these pioneer initiatives in Latin America*.

## RESUMEN

El sobrepeso y la obesidad son una epidemia mundial, en la que se registran tasas que han aumentado hasta niveles alarmantes tanto en los países desarrollados como en los países en desarrollo. Chile no ha sido una excepción, con aumentos pronunciados de la prevalencia de la obesidad, especialmente en los niños en edad escolar. En este documento se describen las políticas y estrategias aplicadas para luchar contra este grave problema de salud pública en Chile durante los 10 últimos años, y se resaltan los principales retos y matices del proceso. Chile ha tomado medidas de política que incluyen el etiquetado frontal de los envases, la reglamentación de la publicidad y restricciones en cuanto a la alimentación en las escuelas. Las nuevas políticas se centran en los determinantes sociales de la salud pues guardan relación con el entorno en cuanto a la alimentación y el comportamiento de las personas. Estas medidas no solo son apropiadas para el contexto actual de Chile, sino que también se basan en las mejores pruebas científicas de que se dispone. Por otro lado, la aplicación de estas políticas ha generado un amplio debate con las instituciones públicas y la industria alimentaria, cuyos temas de discusión abarcan desde derechos de propiedad hasta barreras comerciales. A pesar de algunas diferencias entre los interesados directos, se ha logrado un valioso consenso político y varias organizaciones internacionales están dispuestas a evaluar la repercusión de estas iniciativas pioneras en América Latina.

## RESUMO

O sobrepeso e a obesidade constituem uma epidemia global atingindo níveis alarmantes nos países desenvolvidos e em desenvolvimento. O Chile não é exceção: o país tem registrado uma elevação acentuada da prevalência de obesidade, sobretudo em crianças em idade escolar. Este artigo descreve as medidas envolvendo políticas e estratégias implantadas no Chile na última década para combater este importante problema de saúde pública e destaca os principais desafios e as particularidades do processo. O país adotou políticas para rotulagem nutricional na parte da frente da embalagem dos produtos alimentícios, regulamentação da publicidade e restrições aos alimentos servidos em escolas. As novas políticas são direcionadas aos determinantes sociais da saúde por estarem associados aos ambientes e aos comportamentos alimentares da população. Além de serem adaptadas ao contexto atual do Chile, estas medidas se embasam nas melhores evidências científicas. A execução destas políticas deu início a um amplo debate entre as instituições públicas e a indústria de produtos alimentícios envolvendo de questões sobre direitos de propriedade às barreiras comerciais. Apesar das suas posições divergentes sobre alguns aspectos, os interessados diretos chegaram a um consenso político importante. As organizações internacionais esperam agora conhecer o resultado da avaliação do impacto dessas iniciativas pioneiras na América Latina.

An increasing number of studies from countries around the globe have described a cumulative proportion of the population suffering from overweight or obesity. The World Health Organization (WHO) has warned that in 2008, this epidemic reached catastrophic levels, affecting at least 35% of adults 20+ years of age and causing over 2.8 million premature deaths per year worldwide (1).

The overweight and obesity epidemic is threatening the developed as much as the developing world (2). In countries that pertain to the Organization for Economic Cooperation and Development (OECD), estimates show that 1 in 5 children are either overweight or obese and 18% of adults are obese (3). In Latin American countries, 20% – 25% of the population under 19 years of age (more than 25 million children and 15 million adolescents) are either overweight or obese (4) and almost 1 in 4 adults are obese (5).

Chile was on the same path as prevalence of obesity increased from 23.2% in 2003 to 31.2% in 2017 and overweight increased from 37.8 % to more than 39% during the same time period (6). Moreover, 10.3% of children less than 6 years of age (7) were obese in 2014, as was 1 of every 4 children entering primary school in 2013 (8).

**FIGURE 1.**
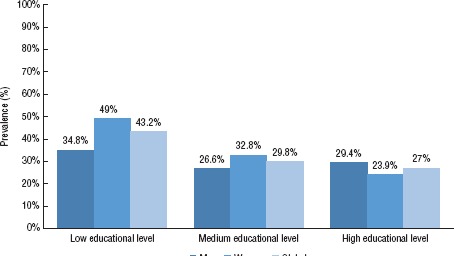
Prevalence of obesity by gender and educational level, Chile, 2017

An additional concern regarding the overweight and obesity epidemic in Chile is its unequal distribution. Obesity prevalence among people with a lower level of education almost doubles its prevalence, compared to those with the highest level of education. Moreover, the unequal distribution is also observed by genre, with a greater proportion of women affected in low and medium educational level, as shown in Figure 1 (6).

This article aims to present different strategies implemented in Chile to reduce overweight and obesity during the last 10 years, discussing the main challenges and barriers observed during the implementation of several policy actions, especially over the last 4 years.

## EVIDENCE TO SUPPORT THE IMPLEMENTATION OF NUTRITIONAL POLICIES

It is well demonstrated that one of the leading causes of overweight and obesity is the high availability of lownutritional and high caloric content food (9). Moreover, intake of this type of food is directly related to an increase in weight (10).

Evidence regarding cost-effectiveness of several nutritional policies, has concluded that three interventions—regulation of food advertising, food labelling, and fiscal measures (such as taxes and subsidies)—would be the most cost-effective (11). Thereby, OECD strongly encourages governments to explore interventions to reduce overweight and obesity, especially those considered in the regulatory and fiscal domain (9).

Evidence shows that Front-of-Package (FOP) labelling systems have an impact on consumers behavior, giving them the chance to make better informed decisions and avoid unhealthy products ((12, 13). Moreover, product labels have long term implications, such as leading product reformulation, creating a new whole market of healthier products (14).

Similarly, evidence regarding food marketing shows that advertisements focus mainly on low nutritional value foods and are aimed primarily at children. Food marketing is capable of attracting children’s attention and enhancing product acceptability (15). Thus, banning unhealthy food advertising shows a positive effect on children’s diets (13).

Likewise, interventions aiming at modifying food environments, such as those in schools, are effective in reducing overweight and obesity in children. Evidence shows that food environments can have a strong influence on the schoolaged population; therefore, interventions at schools could change food preferences, as well as body mass index (BMI) in children (16).

Fiscal measures are also described as effective policies, changing intake behavior ((17, 18) and reducing obesity, though the size of the effect depends on several factors ((19, 20). Systematic reviews mention that although this type of tax could be regressive, it is easily adjustable by adding subsidies to healthy food (such as fruits, vegetables and water), increasing its progressive behavior ((18, 19).

Finally, it is worth highlighting the experience of Mexico, where the prevalence of obesity in 2012 was estimated at 32.7% (21), surpassed only by the United States of the OECD countries (3). As a response to this condition, the Government of Mexico implemented one of the most comprehensives strategies to tackle overweight and obesity. The plan included several measures, such as taxing sugary drinks and high-calorie foods. A big impact of these policies has been anticipated mainly from changing consumption among the population (22).

## POLICY ACTIONS IMPLEMENTED IN CHILE

As the overweight and obesity epidemic is a mayor national public health concern, the Ministry of Health (MoH) of Chile has taken policy actions to control it. Actions carried out focus on two main areas: the social determinants of health related to food environments, and people’s behavior. These actions aim at reducing unhealthy food consumption as much as increasing healthy food consumption.

In 2012, a new regulation (Law 20606) was passed to incorporate a FOP labelling system. This law mandates that foods that exceed certain levels of energy, sugars, sodium, or saturated fatty acids—defined by the MoH—must carry a symbol that warns the potential consumers about its content. Foods that are to be labeled with this symbol are forbidden to be sold in schools and their advertising is strongly restricted.

**FIGURE 2.**
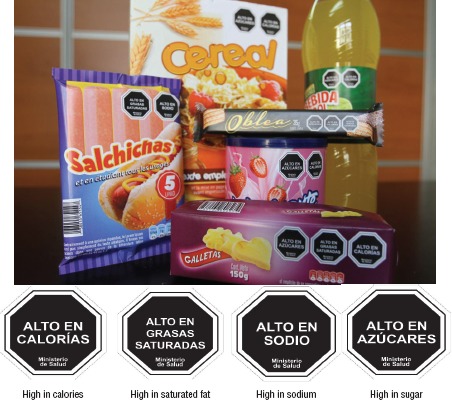
Example of Front-of-Package labels approved by law in Chile, 2015 approved by law in Chile, 2015

Once Law 20606 was passed, the MoH had to establish specific levels of energy, sugars, sodium, or saturated fatty acids, and the characteristics of the symbols to be used—a black octagon (stop sign) as shown in Figure 2. In 2014, important changes were introduced to this regulation to include several unhealthy products that had been excluded previously. In addition, limits used to label a food as unhealthy were adjusted to better impact food environments. Finally, the regulation was made available for public opinion and feedback and took its current form (Box 1).

 These three interventions—FOP labelling, advertising regulations, and school food sales restrictions—follow international recommendations for improving population’s nutritional habits ((23, 24). However, they have been broadly criticized by the food industry that claims these interventions increase consumer barriers to choice and restrict consumer rights. The regulation’s opponents also claim that it interferes with property rights (by restricting industry brands and logos), creates trade barriers, and eliminates the possibility of comparing products, since “all food would be marked as unhealthy.” However, the General Comptroller Office of the Republic of Chile, which is charged with assessing the legality of every government act, has approved every element of this regulation, only introducing small changes to modify the FOP text label from “excess of (nutrient X)” to “high levels of (nutrient X).” Also, many government officials and representatives of civil society have been strong supporters of these changes, providing the necessary political and social backing. The legal process was completed in 2015, but the law’s mandatory implementation phase did not begin until July 2016, giving the food industry ample time to prepare.

Concurrently, public programs have been implemented to promote healthy lifestyles. These programs aim to change people’s behaviors through individual and structural actions taking into account the geography of where they live. For example, the *Vida Chile* (Chile Life) program was started 10 years ago to improve people’s eating and physical activity habits through social communication strategies. Later, the Government launched a program called *Estrategia Global contra la Obesidad* (the Global Strategy against Obesity), which included legislative and regulatory components; and the program *Elige Vivir Sano en Comunidad* (Choose to Live Healthy in the Community) to encourage people to choose healthy food, increase healthy activities in their communities, and recognize the social and geographic element of these choices. Finally, the program *Estrategia Municipios, Comunas y Comunidades Saludables* (Healthy Municipalities, Communes, and Communities Strategy) recognizes local governments as a strategic actor that can create political and strategic conditions that facilitate healthier environments and opportunities for life, valuing the identity of each territory and promoting in its inhabitants a greater level of activity and empowerment regarding their quality of life and well-being. This is also supported by available evidence, showing that behavioral interventions for preventing and treating obesity can have a positive effect on BMI indexes, although impact on physical activity is still unclear (25).

BOX 1. **Details and nuances of the implementation of Law 20-606 in Chile**

This law creates a Front-of-Package label for unhealthy food, defined as exceeding cut-off points in four main nutrients: sugar, calories, sodium, and saturated fats.

The methodology for establishing cut-off points for these four nutrients, considered the intrinsic energy and critical nutrients that each food normally has in its natural form (for example, sugar is not labelled as “high in sugar”). This means that regulation is more focused on food that has critical nutrients added to its natural form. The limits were defined as follows:

**Table:** 

Food type	Cut-off points			
	Energy kcal/100 g	Sodium mg/100 g	Total sugars g/100 g	Saturated fats g/100 g
Solid food	275	400	10	4
Liquid food	70	100	5	3

It is important to mention that this policy is being implemented gradually. The cut-off points listed here will not be used until 2019, following incremental increases from 2016 – 2019. This gradual implementation aims to give the food industry time to develop the technology needed to reduce the content of critical nutrients in their products.

Before the implementation of this law, every Member State of the World Trade Organization was notified through its official contact points, in order to ensure a public consultation for this regulation. More than 3 000 suggestions, observations, and opinions were received, from approximately 350 institutions and individuals. Opinions included declarations supporting this regulation, and suggestions to change or modify different parts of the policy. The regulation was modified based on this public consultation. One of the main observations that was incorporated after the consultation, was the progressive implementation, mentioned above.

***Source:*** Prepared by the authors from study data.

Finally, as a complement to the interventions mentioned previously, the MoH implemented a new fiscal measure to tax sugar-sweetened beverages. This policy aims to reduce sugar consumption in children in order to reduce obesity prevalence in this population group. In addition, a new taxation system for solid food, based on its sugar content, is being explored. This would be a pioneer initiative in Latin America, given that, to date, only Denmark, Finland, and Hungary have such a measure (26).

## CONCLUSIONS

Chile is addressing overweight and obesity issues with several policy measures that are strongly supported by scientific evidence. Despite some disagreements with private industry and other political actors, there has been a dialogue and attitude toward reaching social and political consensus in the context of general welfare and evidence-informed policymaking. The support of key political and civil society stakeholders has been of great importance.

The development of several regulatory interventions addressing social determinants of health and modifying food environments are expected to have a big impact on overweight and obesity. Despite the challenges that will be faced during implementation of Law 20606, evaluation of its nutritional policies would improve understanding of their local impact, augmenting the body of evidence available for decisionmaking. This is particularly important, since most of the evidence presented here is from international studies and may not be completely applicable to the Chilean context.

Finally, although overweight and obesity are well recognized as urgent, global problem, efforts made by single countries are still insufficient, especially among low- and middle-income countries. It is important to collaborate with other nations addressing similar issues, since overweight and obesity are a global concern and joint efforts are more effective than single ones.

### Disclaimer.

Authors hold sole responsibility for the views expressed in the manuscript, which may not necessarily reflect the opinion or policy of the *RPSP/PAJPH* and/or PAHO.
